# A Case of Poisoning by Filix Mas

**Published:** 1897-09

**Authors:** J. Llewellyn Jones

**Affiliations:** Gatesville, Tex.


For the Texas Medical Journal.

### A CASE OF POISONING BY FILIX MAS.

BY J. LLEWELLYN JONES, M. D., GATESVILLE, TEX.

ON THE evening of May 13th, 1897, I was approached by a messenger who appeared to be laboring under great excitement, and who begged that I would go with all dispatch to the home of one J. R.

On arriving at the house I was led to the bedside of a girl, some six years of age, and whose condition seemed serious indeed. Nothing but the faintest fluttering over the region of the heart denoted the existence of any life. The friends already deemed her dead, and filled the house with their lamentations. Feeling that death would certainly ensue if something were not done immediately, I hastily filled the barrel of my hypodermic syringe with a solution of strychnine and atropine sulphate and injected it forthwith. While preparing and administering this stimulant, I was regaled with the following: The girl had been suffering for some two years past with a tape worm, of which her parents were anxious she should be rid. A certain "Lady Doctor," "Magnetic Healer," and "Faith Rubber-in," had volunteered her services. She was to administer the cure, and stay with the patient until results were realized. The results realized were probably not those expected. To make a long story short, she had administered to this six year old child, small for her age, and weakened by thirty-six hours of fasting, in the space of about two hours, a full half ounce of a strong fluid extract of Filix Mas.

The appearance of the child was characteristic—limp, apparently lifeless, eyes open and staring, and the pupils strangely contracted, indeed, the latter were not larger than a pin head, and remained thus contracted until she rallied some four hours later. After a vigorous administration of stimulants—strychnia, atropia, nitroglycerine, ether, and warmth applied to the surface of the body, and just as I was beginning to despair, reaction happily set in, and I had the satisfaction of seeing our little patient "come to." It was but for a short time however, as a violent gastro-enteritis set in, which came very near carrying her off, and it was not until many days later she could be pronounced out of danger.

The story told by the tape-worm doctor was this: She had been in the habit of using an emulsion of male fern for the expulsion of tape worms, but had not been successful in all cases, and so concluded to try the fluid extract which she had been informed was a stronger preparation. She learned a useful lesson in this instance, however, and has since retired, as she declares, permanently from the business.

In connection with the following we might add that Filix Mas is by no means a safe drug in the hands of the ignorant; numerous deaths are reported as resulting from its careless administration, and perhaps it would be wiser for us to select the more insoluble oleoresin for anministration, which expends its action on the mucous membrane of the intestines (where dwelleth ye worm), than the more soluable and highly potent fluid extract which permeates the system freely immediately upon its administration.

I may further add that the girl still passes sections of tænia.

